# Trust Building in Internet-Based Home Care Among Loyal Patients: Qualitative Study

**DOI:** 10.2196/88860

**Published:** 2026-04-15

**Authors:** Yan Yang, Fei Lu, Xinxin Wang, Leiwen Tang, Luchen Pan, Yu Zhang, Hongling Sun, Jia Feng, Chenling Zhu, Meijuan Lan

**Affiliations:** 1 The Second Affiliated Hospital of Zhejiang University School of Medicine, 88 Jiefang Road, Shangcheng District, Hangzhou, 310009, China, 86 13757119537

**Keywords:** home care, loyalty, trust, experience, qualitative study

## Abstract

**Background:**

The growing demand for home-based care, driven by rapid population aging, has accelerated the development of internet-based home care. Despite its emerging status, a subset of loyal patients has consistently used internet-based home care with high frequency, showing strong commitment and a willingness to recommend it to others. Understanding their experiences with trust-building among loyal patients is essential to optimize and scale this service model. However, few studies have specifically explored the experience of trust-building among this patient group, leaving a significant gap in the literature.

**Objective:**

This study aims to explore the experiences of trust-building among loyal patients in internet-based home care, guided by the cognitive-affective-conative model.

**Methods:**

A descriptive qualitative design was used. A purposive sampling method was used to select 15 loyal patients in internet-based home care (mean age 65, SD 18.6, range 30-92 y; mean number of care visits 168, range 101-359) in Zhejiang Province, China. Semistructured interviews, informed by the cognitive-affective-conative framework, were conducted between June 2025 and August 2025. Data were analyzed using directed content analysis, facilitated by NVivo 12.0 software (Lumivero).

**Results:**

Three core themes emerged. The first theme, cognitive dimension—building the foundation of trust through rational appraisal—included channels of trusted information, recognition of professional competence, convenience of digital services, and concerns about safety and privacy. The second theme, affective dimension—deepening trust through emotional and cultural connection—involved from professional interaction to “quasi-family” bonds, personalized care to emotional comfort, and filial piety culture as an emotional and trust catalyst. The third theme, conative dimension—translating trust into loyal behaviors within rational limits—underscored trust-driven advocacy and word-of-mouth, willingness to pay as a monetization of trust, engaged participation in service improvement, and trust-based decisions to continue or terminate services.

**Conclusions:**

Patient trust in internet-based home care is shaped by the interplay of cognitive appraisal, emotional connection, and behavioral intention. This study shows that loyalty first originates from a rational understanding of service quality, which generates trust. During the service process, emotional connections and cultural factors, such as filial piety, further enhance this trust, gradually leading to loyalty behaviors based on rational choice, such as frequent use and word-of-mouth promotion. This study demonstrates that loyalty is not only a rational choice based on service quality but also a commitment anchored in long-term emotional relationships, further reinforced by cultural factors like filial piety that embed the services within family values. The cognitive-affective-conative model effectively captures this multifaceted experience. Consequently, moving beyond mere technical use, future development must strategically integrate emotional support and cultural sensitivity to nurture deep, sustainable trust.

## Introduction

The rising worldwide need for long-term care, driven by rapid population aging and a growing burden of chronic diseases, has accelerated the development of internet-based home care [1]. This emerging model uses digital technology to deliver skilled nursing care directly to patients’ homes, thus improving health care accessibility and efficiency [2,3]. By acting as a bridge over distance and mobility gaps, this model shows significant potential for underserved populations, including older adults, individuals with chronic illnesses, and rural residents [4,5]. Following national pilot programs such as the “Internet Plus Nursing” initiative in 2019, China has actively promoted internet-based home care to reduce health care disparities and support aging in place [4,6].

Despite this potential, a widespread lack of public trust remains a critical barrier to large-scale adoption [7,8]. Trust, a multifaceted psychosocial construct, is pivotal in health care as it underpins patient engagement, adherence, and loyalty [9,10]. It encompasses confidence in the reliability, competence, and integrity of a service or provider. Loyalty represents the culmination of sustained trust and comprises both favorable attitudes and consistent behaviors [11]. Loyal patients are thus defined by significant loyal behaviors—characterized by long-term, high-frequency service use alongside a strong propensity for recommendation [11-13]. Extensive quantitative research across traditional health care and commercial domains has consistently shown that trust enhances outcomes such as patient satisfaction, treatment adherence, and repeat patronage [14-16]. Furthermore, qualitative research findings clearly emphasize the need for health care practitioners and public health officials to prioritize trust-building in community care work, because trust fosters patient engagement, enhances treatment cooperation, and strengthens the patient-provider relationship [17].

However, within the novel and rapidly evolving context of internet-based home care, understanding how trust is built becomes even more critical. This model introduces unique dynamics involving digital interfaces, in-home service delivery, and the mediation of care through a platform, which collectively reshape traditional patient-provider interactions and risk perceptions [7,9]. Investigating how trust is built in this specific setting is therefore essential not merely to fill a gap in literature but to generate actionable insights that can optimize service design, enhance patient adoption, and ensure the sustainable integration of digital solutions into long-term care ecosystems. A deep comprehension of the trust-building experience, particularly among those who have demonstrably embraced the service (loyal patients), is crucial for informing strategies that foster wider acceptance, improve care quality, and ultimately realize the potential of internet-based home care to address pressing societal health challenges.

To systematically investigate how trust is built among loyal patients in this novel service model, this study uses the cognitive-affective-conative (CAC) model [18], which, unlike models focusing primarily on cognitive determinants such as the technology acceptance model [19] and the unified theory of acceptance and use of technology [20], offers a more holistic lens by integrating cognitive appraisal with emotional attachment and behavioral loyalty to capture the dynamic evolution of trust. The CAC model is a well-established theoretical framework that explains how attitudes are formed and transformed across 3 sequential dimensions: cognitive (beliefs and knowledge), affective (emotional responses and attachment), and conative (behavioral intentions and actions). It has been widely applied in health technology adoption research, such as studies on middle-aged adults’ usage of health apps [21] and users’ continued engagement with health education videos [22]. As an attitude, trust is defined as a positive evaluation of the reliability and integrity of a service or provider [9,10]. Trust is conceptualized as a dynamic, multistage psychosocial construct that evolves across these 3 interrelated dimensions—from cognitive appraisal to emotional attachment to behavioral commitment [15]. This operationalization aligns with the CAC model’s sequential logic: cognition shapes initial trust, affective connections deepen trust, and conative behaviors actualize and sustain trust.

Guided by the CAC model framework, this study used a descriptive qualitative design to examine the trust-building experiences of loyal patients receiving internet-based home care in Zhejiang Province, China.

## Methods

### Research Design

Descriptive qualitative research is particularly apt for exploring phenomena that have not been extensively researched [23], as it permits a nuanced and detailed depiction of participants’ lived experiences and sentiments without imposing pre-existing theoretical constraints. Given the paucity of studies specifically examining the health care experiences of loyal patients in internet-based home care, this methodological approach was deemed most appropriate. Furthermore, the reporting of this study meticulously adhered to the SRQR (Standards for Reporting Qualitative Research) guidelines (Checklist 1) [24].

### Setting and Sampling

This study used purposive sampling, augmented by a maximum variation sampling strategy to ensure participant diversity across age, gender, educational attainment, and types of services received. Participants were recruited between June 2025 and August 2025 from the “Zheli Nursing” application platform in Zhejiang Province, China.

Inclusion criteria were as follows: (1) being aged 18 years or older, (2) having clinical care needs matching the scope of internet-based home care services (primarily including malignant tumors, chronic illnesses such as diabetes and hypertension, and postoperative care needs such as wound management and stoma care after major surgeries), (3) sustained use of internet-based home care services for a prolonged period (defined as ≥6 months [25,26]), (4) high-frequency service use (assessed as receiving at least 100 service visits between January 2023 and May 2025; this threshold was determined via analysis of user frequency data from the “Zheli Nursing” platform, representing a level near the median usage among platform users), (5) a favorable attitude toward recommending the same health care provider (assessed via phone screening), and (6) cognitive lucidity and the ability to articulate their perspectives accurately.

Exclusion criteria were as follows: the presence of severe cardiac, neurological, or other conditions that would impede participation in interviews.

Sample size determination adhered to the principle of data saturation, wherein recruitment ceased when no new information or themes emerged from successive interviews [27]. A total of 19 eligible patients were initially enrolled. Among them, 3 participants completed preliminary (pilot) interviews, which were conducted solely to test and refine the interview guide; data from these pilot interviews were not included in the final analysis. Subsequently, 1 enrolled participant was hospitalized due to a change in their medical condition and declined to proceed with the formal interview, resulting in withdrawal from the study. Consequently, a final sample of 15 participants completed the in-depth interviews and were included in the data analysis.

### Data Collection

Data collection was performed using semistructured interviews. The preliminary interview guide, developed based on the CAC model and previous literature [15,18,28], was pilot-tested with 3 eligible patients to identify potential issues in wording, question sequencing, or comprehensibility and subsequently revised accordingly. Two methodologists with extensive qualitative research experience independently reviewed and validated the refined interview guide. The final interview guide encompassed the following key areas, mapped to the CAC dimensions. First, in the cognitive dimension, the key areas were How did you first learn about internet-based home care, and what led you to try it? How do you assess the professionalism and skills of the nurses who provide care? What advantages or conveniences do you perceive in using this digital service compared to hospital visits? Have you had any concerns about safety, privacy, or reliability when using the service? If so, how were they addressed? Second, in the affective dimension, the key areas were How would you describe your relationship with the nurses who visit you? Has it changed over time? In what ways, if any, has the service provided emotional support or comfort to you or your family? Does using this service affect the life of you or your family feel about caregiving and family responsibilities? Finally, in the conative dimension, the key areas were What has motivated you to continue using the service over a long period? Have you recommended internet-based home care to others? If so, why and to whom? Under what circumstances would you consider discontinuing the service? What suggestions do you have to improve the service in the future?

Before each interview, researchers introduced themselves, clarified the study’s purpose and procedures, and obtained written informed consent, informing participants that all interactions would be audio-recorded. Researchers worked to build trust with patients. Interviews were conducted in a quiet, private environment selected by the participant, with no other people present. Ten interviews were conducted in participants’ homes, while 5 were conducted via video call. Throughout the interviews, researchers meticulously observed and documented nonverbal cues such as facial expressions and body language, carefully noting emotional shifts and attitudinal changes. Participants were actively encouraged to articulate their perspectives freely. If the responses were ambiguous or required further elaboration, researchers used techniques such as strategic rephrasing of key information or asking probing questions to ensure data completeness and accuracy. Interview durations ranged from 35 to 57 minutes.

### Data Analysis

During the analysis, interview recordings and other non-numeric data were coded, identified, and summarized using Lumivero NVivo 12 qualitative analysis software [29]. This study used a directed content analysis approach [30], guided by the CAC model as its theoretical framework. This method is particularly well-suited for validating or extending existing theoretical frameworks [31]. The analysis was conducted independently by 2 researchers (XW and FL), both of whom have training in qualitative methods. The analytical approach comprised the following steps. First, the analysis used a hybrid deductive-inductive approach. The CAC model provided an initial theoretical framework for deductive guidance, while the researchers remained open to inductive insights emerging directly from the data. Two researchers first independently immersed themselves in the interview transcripts to achieve familiarity. An initial codebook was developed based on the CAC model and relevant literature. Second, to ensure the robustness of the coding scheme, a pilot phase was conducted. Three transcripts were randomly selected and independently coded by the 2 researchers using the initial codebook. Their results were compared, and discrepancies were discussed. This pilot test led to revisions, including refining definitions, adding new codes for uncovered concepts, and clarifying categorization criteria. This iterative process resulted in a finalized codebook. Third, using the finalized codebook, the two researchers independently coded all 15 transcripts. The text was examined line by line to identify meaningful units, which were labeled with corresponding codes. New codes were created inductively when necessary. Related codes were then grouped based on their conceptual relationships and properties to form broader categories and subcategories. Fourth, the categorized data were further abstracted and organized to identify overarching themes that captured the core patterns relevant to the research questions. Data collection and analysis were conducted concurrently until thematic saturation was achieved, meaning that no new codes or themes emerged from the final interviews. Finally, throughout the analysis, negative case analysis was actively used. The researchers consciously sought out and interrogated data that contradicted or nuanced the emerging themes, ensuring that the findings accounted for disconfirming evidence and reflected the full complexity of participants’ experiences.

This rigorous, iterative process resulted in a structured coding tree (Multimedia Appendix 1). The coding process and its relationship to the subthemes and overarching theme are exemplified in the coding tree (Textbox 1).

Textbox 1. **Example of a coding tree.**

**Raw data**

***P3:** Before being discharged, I worried about how to manage my wound at home. The doctor told me I could book a nurse for home dressing changes, and I immediately felt relieved.*

*My wound is both deep and extensive, and compounded by my diabetes, its management is considerably challenging.*

*The visiting nurse demonstrates exceptional professionalism; she specializes in treating such cases at the hospital.*

*One time, Nurse Mu rushed back from out of town to help me with my wound. She was 5 hours late due to traffic, but I was willing to wait for her just like I would for my daughter.*

**Meaning-bearing content**

- Information from trusted health care providers reduces anxiety and builds initial trust.
- Recognition of nurses’ specialized skills and professional competence builds trust.
- Strong personal commitment to specific nurses reflects deep trust bonds.

**Code**

- Trusted information from clinicians
- Recognition of professional competence
- Patient patience and understanding

**Subtheme**

- Channels of trusted information
- Recognition of professional competence
- From professional interaction to “quasi-family” bonds

**Overarching theme**

- Cognitive dimension—building the foundation of trust through rational appraisal.

### Quality Control and Research Rigor

The rigor of this qualitative study was comprehensively ensured across 4 key dimensions: credibility, dependability, confirmability, and transferability [32,33].

All interviews were conducted by the first author in collaboration with a clinical nurse. Both were female, held master’s degrees in nursing, and were trained in qualitative methodologies. The first author, with extensive experience in nursing management, actively promoted home care initiatives. During data analysis, 2 researchers (XW and FL) independently coded all transcripts and conducted multiple comparative analyses and discussions until consensus was reached. If consensus could not be reached, a third senior researcher was consulted to arbitrate and make the final decision. The refined themes and subthemes were presented back to the participants for member checking (ie, validating the researchers’ interpretation with participants) to confirm their resonance with the lived experiences. Participants did not raise any objections to the results. To strengthen dependability, all interview recordings were transcribed verbatim independently by the 2 researchers within 24 hours postinterview, followed by cross-verification of the transcribed texts against the original audio. In instances of ambiguous phrasing or incomplete information during transcription, researchers promptly contacted participants for clarification or conducted supplementary interviews to ensure data accuracy and completeness. Regarding confirmability, the research team maintained continuous reflective journals throughout the data collection and analysis phases. These journals focused on critically examining aspects such as adequate respect for participant perspectives, avoidance of personal assumptions, leading language and potential biases, appropriateness of analytical methods, vigilance against the omission of crucial information, and the potential influence of these factors on data interpretation. The refined themes and subthemes were presented back to the participants for member checking to validate their resonance with the lived experiences. Participants did not raise any objections to the results. Finally, the transferability of the study findings was ensured through a comprehensive and detailed description of the research context, participant sociodemographic characteristics, and methodological procedures.

### Researcher Reflexivity and Positionality

The primary researchers hold master’s degrees in nursing and are actively engaged in promoting home care initiatives. This professional background provided deep insider knowledge and facilitated rapport with participants, likely encouraging richer discussions about care experiences. However, this same positionality carried a potential risk of pro-innovation bias—a subconscious tendency to view the service positively or to overlook its shortcomings. To mitigate this, we implemented several safeguards. First, the interview guide was designed with open, neutral questions that explicitly asked about challenges and negative experiences. Second, data analysis was conducted independently by 2 researchers who were not involved in data collection, bringing a degree of outsider perspective to counteract potential collection biases. Third, negative case analysis was deliberately applied throughout the analytic process to identify and interrogate data that contradicted or refined the emerging themes of positive trust-building. This reflexive practice aimed to balance the benefits of insider understanding with disciplined critical distance throughout the research process.

### Ethical Considerations

Ethical approval for this study was obtained from the medical ethics committee of the Second Affiliated Hospital of Zhejiang University School of Medicine (2025‐1196). Researchers rigorously adhered to the principles of voluntary participation and participant confidentiality. Formal interviews were initiated only after participants had provided written informed consent. Participants did not receive any form of compensation for their involvement in this study. To safeguard personal identity information, all participants were assigned unique identifiers (eg, P1, P2, and so on), which were used in interview records and throughout the analysis. Any incidental mentions of third parties within the interview transcripts were redacted before data analysis. Access to raw data or unredacted materials was strictly restricted to the research team.

## Results

### General Characteristics of Participants

A total of 15 loyal patients in internet-based home care participated in this study (Table 1). Patients ranged in age from 30 to 92 years, with a mean age of 65 (SD 18.6) years. The home visits they received ranged from 101 to 359, with an average of 168. In terms of education, 46.7% (n=7) of patients had a secondary school education. The vast majority of care (n=14, 93.3%) for patients was provided by family members.

**Table 1.** General characteristics of interviewees (N=15).

Participant	Sex	Age (y)	Education level	Primary caregiver	Primary diagnosis	Key nursing procedures	Number of visits	Same nurse^a^
P1	Male	60	Higher education	Care worker	Paraplegia and pressure injury	Pressure injury care	359	Yes
P2	Male	92	Primary education	Offspring	Pressure injury, diabetes, and hypertension	Pressure injury care	146	Yes
P3	Male	72	Secondary education	Offspring	Heatstroke, hypertension, and diabetes	Chronic difficult wound	104	Yes
P4	Female	73	Uneducated	Offspring	Postcolorectal cancer surgery	Wound care and ostomy care	101	Yes
P5	Female	41	Secondary education	Spouse	Malignant tumor	Chronic wound and lymphedema care	103	Yes
P6	Male	76	Primary education	Offspring	Postliver cancer surgery	Pain management, drain bag change, and venipuncture	105	Partially
P7	Female	64	Primary education	Offspring	Postmalignant tumor surgery, chronic heart failure, and nephropathy	Subcutaneous injection, PICC^b^ care, and drain bag change.	292	No
P8	Female	86	Secondary education	Offspring	Stroke, hypertension, and diabetes	Pressure injury care, bladder irrigation, and blood glucose monitoring.	154	Yes
P9	Female	33	Higher education	Parents	Postfracture surgery	Wound care and pain management	151	Yes
P10	Female	72	Uneducated	Offspring	Malignant tumor	PICC care and pain management	170	Yes
P11	Female	87	Secondary education	Offspring	Cerebrovascular accident, hemiplegia, and hypertension.	Nasogastric feeding care, catheter care, and subcutaneous injection.	114	No
P12	Female	67	Secondary education	offspring	Stroke, hypertension, and diabetes	Pressure injury care, catheter care, and nasogastric feeding care	163	Yes
P13	Female	50	Secondary education	Spouse	Malignant tumor	PICC care and pain management	110	No
P14	Female	69	Secondary education	offspring	Malignant tumor	Venipuncture, infusion port care, and pressure injury care	158	Yes
P15	Female	33	Higher education	Spouse	Pregnancy and threatened abortion	Subcutaneous or intramuscular injection, venipuncture, and neonatal care	292	No

a Same nurse: whether the patient designates the same nurse for every visit.

b PICC: peripherally inserted central catheter.

Primary diagnoses of patients predominantly included malignant tumors, chronic illnesses, and post–major surgical conditions. The 36 distinct nursing services provided were grouped into specialized and general procedures based on their operational complexity. The performance of nursing procedures is strictly governed by staff qualifications, which are defined by operational complexity. For specialized procedures such as complex wound management and implantable port care, provincial or higher specialized certifications or senior titles are mandated. For general procedures, including subcutaneous injections and urinary catheter changes, the criteria are an intermediate title coupled with a minimum of 5 years of clinical experience.

**Figure 1.**
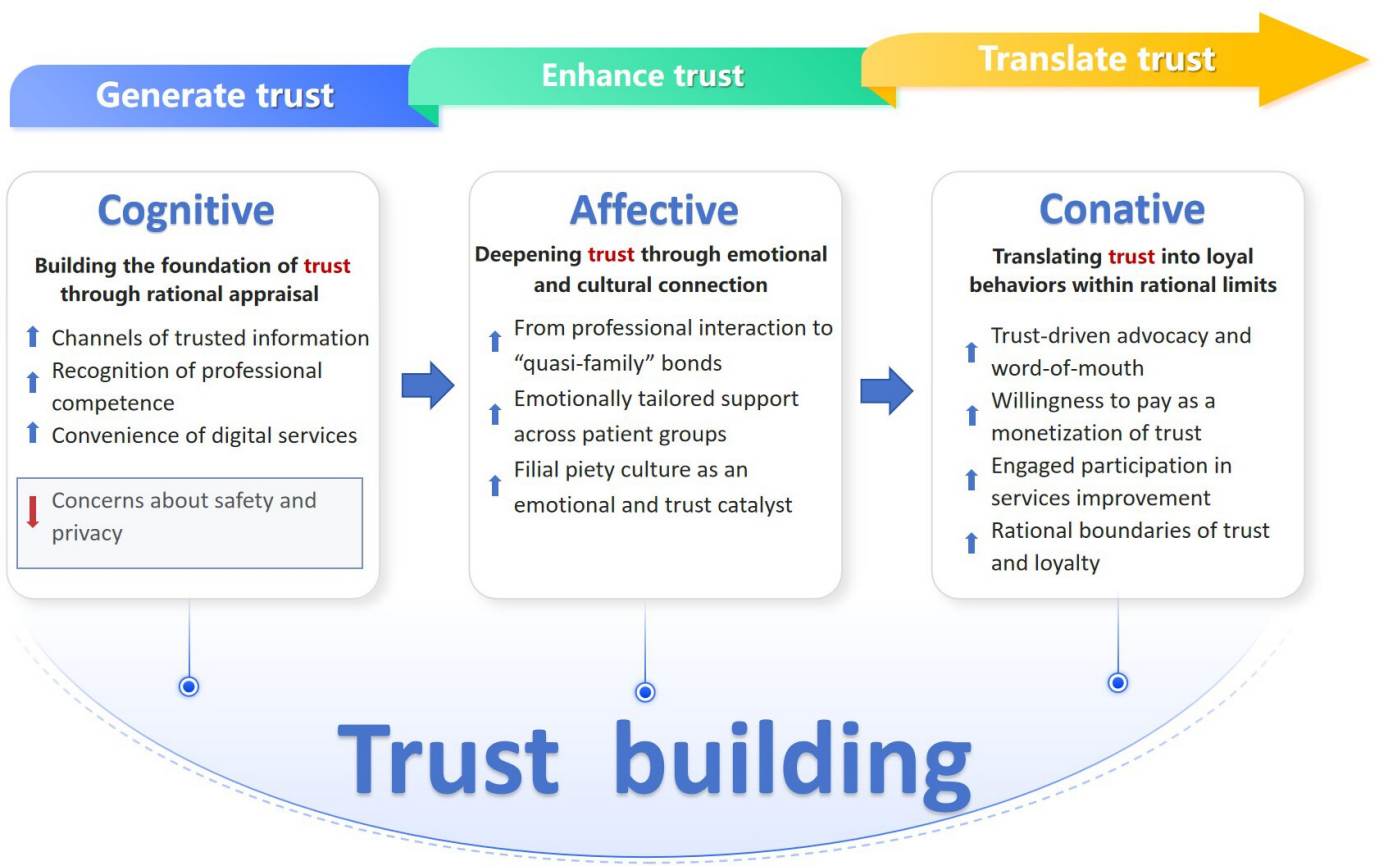
The hierarchical diagram of the 3 main themes and their associated subthemes.

The interviewed loyal patients shared their views on the experiences and drivers that build trust. From the interviews, 3 main categories emerged (Figure 1). It is worth noting, however, that when talking about direct care, all informants in this study referred to nurses collectively. The term “staff,” without distinguishing between nurses with different educational levels, was used, and the patients carefully avoided mentioning any names. Only when talking about social relationships with the nurses did a few patients use the first name of a specific nurse with whom they had a closer relationship.

### Cognitive Dimension: Building the Foundation of Trust Through Rational Appraisal

Within this category, 4 subcategories were generated: channels of trusted information, recognition of professional competence, convenience of digital services, and concerns about safety and privacy, reflecting patients’ cognitive assessment of internet-based home care at the rational level, which form the basis for trust and loyalty.

#### Channels of Trusted Information

The process of building trust in Internet-based home care began with patients becoming aware of the services, with the information source playing a critical role in shaping their initial level of confidence. Participants consistently reported that information from interpersonal and professional channels—such as recommendations from treating clinicians during hospital discharge or from friends and family with direct experience—was paramount in alleviating anxieties and building foundational trust. These trusted sources acted as a powerful validation of the services' reliability and safety. In contrast, impersonal channels, like media advertisements, were effective for raising general awareness but often required reinforcement from personal networks to translate awareness into trust:

*Before being discharged, I worried about how to manage my wound at home. The doctor told me I could book a nurse for home dressing changes, and I immediately felt relieved.*

[P3]

*A friend recommended I use my phone to book a nurse for injections at home, saying his father used it and it was great. Actually, I had seen advertisements on WeChat videos before, but I didn't take it seriously until a friend recommended it; then I tried it.*

[P5]

#### Recognition of Professional Competence

Interviewed patients consistently expressed confidence in the nurses’ ability to manage their health issues at home. The professional competence of the nursing staff was emphasized as a fundamental driver in building initial trust and encouraging continued use of services. This reliance was particularly evident among patients with complex or high-risk care needs, who showed a strong preference for nurses with specialized technical skills:

*My wound is both deep and extensive, and, compounded by my diabetes, its management is considerably challenging. The visiting nurse demonstrates exceptional professionalism; she specializes in treating such cases at the hospital.*

[P3]

*The infusion port is a new device, and general nurses usually don’t know how to handle it. I always ask nurse Huang to come to my home.*

[P14]

#### Convenience of Digital Services

Patients reported that the services significantly reduced the time and effort associated with hospital visits, highlighting the convenience of receiving care at home. The platform’s ability to offer a wide range of choices was also frequently mentioned. Even older adult patients, who faced digital barriers, experienced this practical benefit with caregiver assistance, which reinforced their trust and willingness to continue using the services:

*I have bone metastasis from breast cancer, and I need to go to the hospital 2 times a week. It used to require an ambulance and two family members to accompany me. Now that nurses come to my home, it’s not only convenient for my family, but I no longer need an ambulance.*

[P5]

*Booking on my phone is very convenient. I can choose the hospital, nurse, and time based on my situation, and I can even book several services together.*

[P7]

*I’m old and don't know how to use a smartphone, but my daughter handles the booking. She says it’s very easy to just click a few buttons on the phone.*

[P4]

#### Concerns About Safety and Privacy

Some patients reported concerns regarding the reliability, safety, and privacy protection of services during their initial use. While trust in government-endorsed platforms and transparent information could alleviate some safety doubts, perceived risks of privacy breaches during actual service delivery remained a potential hurdle to the full establishment of trust:

*My wife is concerned about the safety of strangers coming into our home. But I’m not worried because I booked through the Provincial Health Commission’s internet hospital platform and chose a nurse from a well-known hospital.*

[P6]

*Once, when the nurse was here, my wife and daughter-in-law argued. I felt very awkward and worried the nurse would talk about it outside, spreading our “family shame.”*

[P6]

### Affective Dimension: Deepening Trust Through Emotional and Cultural Connection

In this category, 3 subcategories emerged: from professional interaction to “quasi-family” bonds, personalized care to emotional comfort, and filial piety culture as an emotional and trust catalyst, revealing the emotional dependencies and cultural drivers formed by loyal patients during long-term service interactions, which are pivotal in transforming initial trust into deep-seated trust and loyalty.

#### From Professional Interaction to “Quasi-Family” Bonds

Patients described how, over multiple visits and as their health needs were consistently met, their relationships with nurses gradually deepened beyond a purely professional dynamic. Many recounted developing strong personal trust with their nurses, often using family-like terms to describe these bonds. In their narratives, patients highlighted small, personal gestures and emotional support that made them feel cared for beyond the clinical procedures. This sense of personal connection contributed significantly to their overall trust in the services. The strength of these relational bonds was also evident in patients’ willingness to accommodate unusual circumstances. Some expressed a high degree of patience and understanding when service delays occurred, reflecting the depth of their personal commitment to specific nurses:

*Nurse Zhang is like a member of my family. She encourages me, and even brings local snacks during holidays. I’ve been bedridden for 37 years, and I’m most afraid of pressure injuries, but with her these past 2 years, I feel at ease.*

[P1]

*One time, nurse Mu rushed back from out of town to help me with my wound. She was 5 hours late due to traffic, but I was willing to wait for her just like I would for my daughter.*

[P3]

*Nurse Li is like my daughter. She is professional in wound care, chats with me.*

[P2]

#### Personalized Care to Emotional Comfort

The pathways through which emotional connection deepened trust varied significantly among patients, closely aligned with their specific health conditions and corresponding care expectations. Participants with complex, high-risk needs—such as difficult wound management—associated emotional security directly with the nurse’s specialized technical competence. The assurance that a highly skilled professional was managing their care provided them with substantial reassurance. In contrast, for patients requiring routine procedures, such as catheter changes, emotional comfort arose primarily from the service’s overall reliability and convenience. Consistent and accessible care alleviated their anxiety, rendering the specific identity of the nurse less important. Younger patients, including new mothers, derived emotional comfort from the efficiency of the service and its seamless integration into their daily lives, which contributed to a practical sense of ease:

*My complex wound can't be handled by ordinary nurses. I have to ask nurse X to help me—her skills are that good.*

[P3]

*I need regular catheter changes and enteral feeding tube care. For these, as long as a nurse can come to my home to handle these issues, that’s fine—convenience is key, and it doesn't matter who comes. However, when it comes to wound care, I want a specialist nurse to do it.*

[P8]

*Having a nurse come to my home for injections has been fantastic. It means I can avoid hospital trips entirely and have real peace of mind during my pregnancy. After my baby was born, I booked neonatal home care, so convenient.*

[P15]

#### Filial Piety Culture as an Emotional and Trust Catalyst

Some patients frequently described how internet-based home care helped alleviate feelings of being a burden on their families, with their narratives often highlighting a sense of relief as the services not only addressed their clinical needs but also provided a modern solution to the challenge of long-term care—enabling their offspring to fulfill filial duties despite practical constraints, in keeping with traditional beliefs. The motivation of their offspring to book these services was often interpreted by patients as a direct expression of care and filial devotion, strengthening their trust in both the services and their family relationships. For families with members abroad, the services were portrayed as a vital digital bridge. Overseas offspring could actively participate in their parents’ care remotely, transforming physical absence into engaged, thoughtful involvement and easing anxieties on both sides:

*Every hospital visit in the past meant my offspring had to take turns using their precious leave to accompany me. It was hard on their jobs. The guilt would weigh on my conscience. Now, with the nurses coming, no one needs to take leave. Everyone is so much happier.*

[P11]

*My offspring don't have the time to be constantly present, nor the specialized skills needed, like changing wound dressings. Now, with home-based nursing care, this issue is beautifully resolved.*

[P8]

*My daughter insisted on choosing the services for me. She was worried about hospital infections.*

[P4]

*My offspring are abroad, I'm lying at home. They are very filial, although they can't be here to look after me. I don't talk much about my condition for fear of worrying them, but that just makes them worry more. Now, they book nurses for me online. Every time a nurse visits, they join a video call and talk with her about my health. They feel much more at ease abroad.*

[P11]

### Conative Dimension: Translating Trust Into Loyal Behaviors Within Rational Limits

The third theme captured how trust is behaviorally enacted, focusing on the conative dimension of patient loyalty. Subcategories included trust-driven advocacy and word-of-mouth, willingness to pay as a monetization of trust, engaged participation in service improvement, and trust-based decisions to continue or terminate services.

#### Trust-Driven Advocacy and Word-of-Mouth

Most interviewed patients expressed a strong willingness to engage in trust-driven word-of-mouth dissemination. Their recommendations were often directed toward other patients and families in similar health situations, highlighting a pattern of advocacy rooted in shared experiences and the recognized value of services:

*If there are long-term bedridden patients who need care, I will recommend it to them.*

[P9]

*Internet-based home care is especially beneficial for patients discharged after major surgery with weakened immunity. It saves them from going to the hospital and reduces cross-infection. I will recommend it to these people.*

[P4]

#### Willingness to Pay as a Monetization of Trust

A clear willingness to pay a premium for the services was expressed by many patients, even when acknowledging the higher costs compared to in-hospital care and limited insurance coverage. In explaining their stance, participants frequently framed their decision not primarily in terms of financial cost, but through the lens of the overall value received—weighing professional competence, relational continuity, and emotional support against the monetary outlay:

*Each visit costs 2 to 3 hundred yuan, and after over 100 visits, the financial pressure is not small, but I still insist on choosing it.*

[P10]

*Although it’s more expensive than the hospital, compared to the cost of an ambulance and my family’s lost work time, it’s still more cost-effective.*

[P5]

#### Engaged Participation in Services Improvement

Participants provided substantive suggestions for service improvement, reflecting a proactive engagement with the internet-based home care platform. Their input frequently addressed perceived barriers to wider adoption, such as cost, and extended to visionary proposals for enhancing care delivery. These included aspirations for multidisciplinary team visits and the integration of advanced technologies to support aging in place:

*I hope it can be covered by basic medical insurance, reimbursing part of the cost, or by commercial insurance for full reimbursement.*

[P8]

*I hope ultrasound can come to the home; doctors, rehabilitation therapists, and nurses can all visit together.*

[P1]

*In the future, smart monitoring equipment can be rented, with data linked. When we are recovering or aging at home, it can intelligently remind us to eat, take medicine, and exercise. And give me a robot to talk to me and to push my wheelchair when I want to go out.*

[P10]

#### Trust-Based Decisions to Continue or Terminate Services

Participants described clear boundaries to their use of internet-based home care, delineating the conditions under which their trust and loyalty would rationally apply. Their continued reliance was contingent upon the services’ capacity to safely and effectively meet their clinical needs at home. Once those needs evolved beyond the scope of home-based care or were resolved entirely, patients acknowledged they would discontinue the services, indicating a pragmatic and context-dependent dimension to their trust:

*Unless my condition changes and I must be hospitalized, otherwise I will continue to choose it.*

[P4]

*Once fully recovered and mobile, I won't need nurses to come home.*

[P9]

## Discussion

To our knowledge, this study is the first to investigate how trust is built among loyal patients in internet-based home care, guided by the CAC model. Our findings reveal that trust develops through a dynamic, multistage process: it is initially formed through cognitive appraisal of the service’s use and professionalism, deepened via affective and cultural connections, and ultimately enacted through conative behaviors that reinforce and sustain loyalty.

### Principal Findings

The foundation of trust is established within the cognitive dimension, where patients conduct a rational evaluation of the services’ core value and reliability. Consistent with the CAC model’s premise that cognition forms the basis of attitude [18], our findings confirm that trust originates from logical appraisals. First, access to trusted information channels—particularly interpersonal recommendations from treating clinicians or family members—serves as a critical initial trigger. This aligns with prior research informed by perceived risk theory, which emphasizes that information from credible sources significantly reduces uncertainty and facilitates initial trust formation [34]. Our results further substantiate that, unlike impersonal channels such as advertisements, interpersonal endorsements provide essential social validation that enhances trustworthiness, corroborating earlier findings on the primacy of relational cues in health services adoption. Second, the recognition of professional competence—especially in managing complex care needs such as difficult wounds—constitutes a cornerstone of cognitive trust. This observation resonates with French and Raven’s [35] bases of social power, particularly the concept of expert power, wherein trust is grounded in perceived knowledge and skill. Our data reinforce prior studies indicating that patients’ reliance on nurses with specialized certifications reflects a non-negotiable expectation of professional credibility in health care contexts—a consistent theme across trust-related literature in both digital and conventional care settings. Third, the convenience afforded by digital services—including reduced travel burden and flexible scheduling—was shown to enhance perceived usefulness and ease of use, core constructs of the technology acceptance model [19]. This finding is consistent with earlier work on technology-mediated care, which highlights practical use as a key facilitator of trust. However, our study also reveals that this nascent trust remains fragile and can be undermined by persistent concerns about safety and privacy. This echoes prior research underscoring the need for systemic safeguards, such as government endorsement and transparent service protocols [36], to solidify cognitive trust—a recurring recommendation in studies on digital health adoption.

Moving beyond rationality, trust is profoundly deepened in the affective dimension through relational and cultural elements. The evolution of nurse–patient relationships from professional interactions to “quasi-family” bonds is pivotal. This transition, characterized by emotional companionship and personalized care, fulfills patients’ needs for belonging and emotional security, resonating with the principles of emotional social support theory [37]. Such emotional bonds are particularly crucial for elderly or chronically ill patients, as they mitigate feelings of isolation and transform the service into a source of psychosocial sustenance—thereby deepening trust beyond mere transactional satisfaction. These findings align with prior qualitative studies in long-term and home-based care, which similarly identify emotional support and personalized interaction as key mechanisms for sustaining patient engagement and trust [15,16]. Furthermore, this study highlights the underexplored role of filial piety culture as an emotional and trust catalyst. In a societal context where adult offspring often struggle to provide hands-on care due to geographic or professional constraints, internet-based home care offers a culturally congruent solution. It enables offspring to discharge their filial duties by procuring professional care, thus embedding the service within a valued cultural narrative [38]. This cultural resonance generates a powerful, affect-based trust that is particularly potent in the Chinese context. Our results corroborate and extend recent discrete choice experiments indicating that Chinese patients and families highly value home-based care models that align with filial norms [37]. This demonstrates how trust is built not only through service quality but also through alignment with deeply held cultural values—a dimension less emphasized in Western-centric trust models. Importantly, the underlying mechanisms illuminated by this culturally specific finding may hold broader relevance. The core idea—that trust is deepened when a service resonates with users’ fundamental relational values and emotional needs—is likely generalizable. In Western contexts, different cultural or social values (eg, autonomy, independence, and community support) might serve as analogous trust catalysts [39]. For instance, a home care service that effectively supports an older adult’s desire to maintain independence or that strengthens their connection to a local community could generate similarly powerful affective trust [40]. Therefore, while the specific cultural artifact (filial piety) is context-bound, the broader theoretical implication is that trust-building strategies must identify and align with the core relational and emotional value systems of the target population.

The conative dimension represents the behavioral manifestation of trust. Loyal patients do not merely hold trust internally; they enact it through tangible behaviors. Trust-driven advocacy and word-of-mouth are direct translations of trust into promotion. Patients become voluntary ambassadors, primarily recommending the services to peers in similar situations—a behavior rooted in empathy and shared experience. This aligns with Dick and Basu’s [11] conceptualization of loyalty, wherein a positive attitude leads to supportive behavioral intentions, and extends prior findings that recommendation intent is a core component of attitudinal loyalty in health care settings [14]. Similarly, the willingness to pay a premium, despite limited insurance coverage, demonstrates the monetization of trust. Patients conduct a cost-benefit analysis, as suggested by expectation-confirmation theory [41], and their willingness to pay reflects a holistic valuation of the trust they have developed—encompassing both cognitive and affective benefits. This finding resonates with economic loyalty models that frame price tolerance as a function of perceived value and relational investment [10], yet it further specifies that in home care, this valuation integrates clinical competence with emotional and cultural use. Moreover, patients’ engaged participation in service improvement signals a high level of trust and co-ownership. This proactive engagement indicates that trust has fostered a partnership dynamic, where patients feel invested in the service’s success. Such behaviors reflect principles of co-production often discussed in service-dominant logic and patient-centered care literature [14,16], but our study situates them within the digital home care context as outcomes of accumulated trust. Crucially, this study also identifies the rational boundaries of trust and loyalty. Trust is not blind. Patients’ loyalty remains contingent on the service’s ability to meet their clinical needs safely and effectively. This rational boundary underscores a mature and context-dependent form of trust, ensuring that the trust-building process remains grounded in realistic expectations of the service’s scope. This nuanced view challenges the sometimes static portrayals of loyalty in earlier literature [42], highlighting its conditional and dynamic nature in ongoing service relationships.

### Theoretical and Practical Implications

In synthesis, the process of how trust is built in internet-based home care is a reinforcing cycle: cognitive assessments establish its foundation, emotional and cultural connections deepen its roots, and conative behaviors both manifest and reinforce it, creating a positive feedback loop that sustains trust and loyalty. This dynamic aligns with and extends the CAC model [18], while also resonating with social exchange theory in relational contexts, where reciprocal reinforcement between trust and behavior has been widely observed [11]. This study’s identification of filial piety as a distinct emotional and trust catalyst extends the application of the CAC model in digital long-term care in two significant ways. First, it demonstrates the model’s contextual adaptability, showing how a core cultural value permeates all 3 trust-building dimensions. Filial norms influence cognitive appraisals (eg, interpreting offspring’s service booking as an act of care), fuel affective bonds by transforming professional care into “quasi-family” relationships, and motivate conative loyalty as acts of family duty [38]. Thus, filial piety acts as an active, culturally embedded force that strengthens the entire CAC sequence. Second, and more importantly, it refines the model’s affective dimension by specifying a powerful, culturally shaped source of emotional attachment—thereby revealing a generalizable mechanism. While the CAC model acknowledges that emotions deepen trust [18], it often treats their origins as individual, idiosyncratic, or vague. Our findings demonstrate that affective responses can be systematically rooted in shared normative values that function as collective motivators [38]. This theoretical insight moves beyond the specific case of filial piety; in any cultural context, trust is deepened when a service resonates with users’ fundamental relational values and emotional needs [39]. This mechanism is culturally universal, though its manifestations are context-specific. In China, filial piety serves as the dominant cultural frame; in Western contexts, analogous trust catalysts may include autonomy and independence (eg, services that enable older adults to age in place without relying on family), community connectedness (eg, services that integrate home care with local social networks), or reciprocity and dignity (eg, models that frame care as partnership rather than dependency) [40,41]. Consequently, the CAC model evolves from a general psychological sequence into a culturally informed framework that can better predict which emotional drivers will be effective in specific settings.

For practice, this multifaceted understanding demands integrated strategies that are both universally applicable and culturally adaptable. To build cognitive trust, platforms must ensure information transparency, showcase nurse credentials, optimize usability, and implement robust safety and privacy protocols. These measures directly address the cognitive drivers identified in this study and are consistent with prior recommendations for mitigating perceived risk in digital health services [34,36]. To foster affective trust, service models should encourage relational continuity (eg, through the same nurse assignment) and train nurses in empathetic communication and emotional support—strategies that are effective across cultures. However, the cultural framing of marketing and service narratives must be locally tailored. In China, highlighting the service’s role in enabling filial piety has proven powerful [38]; in other contexts, promotional messages might emphasize preserving independence, strengthening community ties, or upholding personal dignity. The common principle is not the specific value used, but the strategic alignment with what patients and families culturally hold dear. To facilitate conative trust, providers should create structured channels for patient feedback and co-design, reflecting the partnership dynamic observed in our data. Meanwhile, policymakers should explore insurance reforms to reduce financial barriers, thereby validating patients’ willingness to invest in a trusted service—a finding supported by both our results and economic models of loyalty [10,41]. These integrated strategies not only respond to the multi-stage trust-building process identified in this study but also align with emerging calls in the literature for more culturally and emotionally informed approaches to digital home care implementation [2,6,38].

### Limitations

There are various limitations to consider when interpreting the results. First, the operational definition of “loyalty” is based on a specific behavioral threshold and participants’ self-reported word-of-mouth recommendation intent. Future research would benefit from incorporating multidimensional, objective behavioral indicators, such as frequency, duration, and cross-temporal continuity of use, for more robust validation, or could further explore the robustness of findings under different operational thresholds to define and measure patient loyalty more comprehensively. Second, during data collection, requiring participants to retrospectively recount their experiences with internet-based home care may have introduced recall bias. The scope of this study’s “loyal customer” interview sample was restricted to Zhejiang Province, China. Given the substantial regional variations in economic conditions, cultural contexts, and health care resources across China, these geographic limitations may impact the generalizability of our findings. Third, by focusing exclusively on loyal patients, this study offers deep insight into the processes of trust consolidation but does not capture experiences of trust erosion, dissatisfaction, or service discontinuation. Consequently, our understanding of trust dynamics remains partial. Future research should explicitly address this gap by incorporating comparative designs, for example, interviewing nonloyal users, former users who discontinued the service, or those who experienced trust violations, to provide a more holistic view of trust formation, maintenance, and attrition in internet-based home care. Finally, while qualitative research offers rich, in-depth insights, the generalizability of its conclusions warrants further substantiation. We recommend that future studies develop standardized scales and integrate quantitative methods to rigorously analyze the influence pathways and weights of the cognitive, affective, and conative dimensions on loyal behavior. Subsequent research should also broaden the sample scope and conduct multicenter studies nationwide to comprehensively and profoundly investigate variations in internet-based home care experiences regarding their trust across diverse populations.

### Conclusions

This study, grounded in the CAC model, is the first to systematically reveal the dynamic process of trust-building among loyal patients in internet-based home care. The findings indicate that trust formation originates from a rational cognitive appraisal of information channels, professional competence, service convenience, and safety and privacy concerns. Through sustained interactions, nurse-patient relationships evolve into “quasi-family” bonds, and under the influence of filial piety culture, emotional connections deepen, embedding trust within both affective and cultural dimensions. Ultimately, trust translates into loyal behaviors, including continued use, word-of-mouth advocacy, willingness to pay, and engaged participation in service improvement, all bounded by rational considerations of clinical need and safety.

The originality and contribution of this study are threefold. Theoretically, it extends the CAC model to the context of digital home care and identifies “filial piety culture” as a key emotional and trust catalyst, enhancing the model’s explanatory power and cultural adaptability. Empirically, by focusing on the under-researched group of loyal patients, it maps the complete trajectory of trust evolution from cognitive appraisal through affective deepening to behavioral enactment, offering a novel framework for understanding sustained trust in digitally mediated care. Practically, the findings provide clear guidance for service optimization: strengthening nurses’ dual professional and emotional competencies, implementing “same-nurse” systems, improving platform usability, enhancing privacy protection, promoting insurance coverage, and aligning service design with cultural values such as filial piety to support families in fulfilling care responsibilities.

In conclusion, through its theoretical, empirical, and practical contributions, this study offers a theoretically grounded and actionable roadmap for building a trust-driven, patient-centered digital home care ecosystem in aging societies.

## Supplementary material

10.2196/88860 Multimedia Appendix 1 Complete coding tree.

10.2196/88860 Checklist 1 SRQR checklist.

## Abbreviations

CAC: cognitive-affective-conative
SRQR: Standards for Reporting Qualitative Research
