# Supplementary material for: Trust Building in Internet-Based Home Care Among Loyal Patients: Qualitative Study
Source: J Med Internet Res. 2026 Apr 15;28:e88860. doi: 10.2196/88860 (PMC13082572; doi:10.2196/88860)
Supplement: Multimedia Appendix 1 [file jmir-v28-e88860-s001.docx]

**Table 3**. Coding tree

| **Raw data** | **Meaning bearing content** | **Code** | **Sub-theme** | **Overarching theme** |
| --- | --- | --- | --- | --- |
| P3: Before being discharged, I worried about how to manage my wound at home. The doctor told me I could book a nurse for home dressing changes, and I immediately felt relieved.  My wound is both deep and extensive, and compounded by my diabetes, its management is considerably challenging.  The visiting nurse demonstrates exceptional professionalism; she specializes in treating such cases at the hospital.  One time, Nurse Mu rushed back from out of town to help me with my wound. She was five hours late due to traffic, but I was willing to wait for her just like I would for my daughter. | Information from trusted healthcare providers reduces anxiety and builds initial trust.  Recognition of nurses' specialized skills and professional competence builds trust.  Strong personal commitment to specific nurses reflects deep trust bonds. | Trusted information from clinicians  Recognition of professional competence  Patient patience and understanding | Channels of trusted information  Recognition of professional competence  From professional interaction to 'quasi-family' bonds | Cognitive dimension—building the foundation of trust through rational appraisal |
| P5: A friend recommended I use my phone to book a nurse for injections at home, saying his father used it and it was great. Actually, I had seen advertisements on WeChat videos before, but I didn't take it seriously until a friend recommended it; then I tried it.  I have bone metastasis from breast cancer, and I need to go to the hospital two times a week. It used to require an ambulance and two family members to accompany me. Now that nurses come to my home, it's not only convenient for my family, but I no longer need an ambulance.  Although it's more expensive than the hospital, compared to the cost of an ambulance and my family's lost work time, it's still more cost-effective. | Interpersonal recommendations from peers are more effective than impersonal advertising for building trust.  Home-based care reduces burden on patients and families, enhancing trust through practical utility.  Patients conduct holistic cost-benefit analysis valuing trust and convenience. | Interpersonal recommendations  Convenience reduces burden  Holistic value assessment | Channels of trusted information  Convenience of digital services  Willingness to pay as a monetization of trust | N/A |
| P14: The infusion port is a new device, and general nurses usually don't know how to handle it. I always ask Nurse Huang to come to my home. | Patients prefer nurses with specialized technical skills for complex procedures. | Preference for specialized nurses | Recognition of professional competence | N/A |
| P7: Booking on my phone is very convenient. I can choose the hospital, nurse, and time based on my situation, and I can even book several services together. | Digital platform flexibility and choice enhance user experience and trust. | Digital platform flexibility | Convenience of digital services | N/A |
| P6: My wife is concerned about the safety of strangers coming into our home. But I'm not worried because I booked through the Provincial Health Commission's internet hospital platform and chose a nurse from a well-known hospital.  Once, when the nurse was here, my wife and daughter-in-law argued. I felt very awkward and worried the nurse would talk about it outside, spreading our 'family shame'. | Government-endorsed platforms and transparent information alleviate safety concerns.  Privacy concerns during service delivery remain a potential barrier to full trust. | Safety concerns mitigated by platform credibility  Privacy concerns about family matters | Concerns about safety and privacy  Concerns about safety and privacy | N/A |
| P1: Nurse Zhang is like a member of my family. She encourages me, and even brings local snacks during holidays. I've been bedridden for 37 years, and I'm most afraid of pressure injuries, but with her these past two years, I feel at ease.  I hope ultrasound can come to the home, doctors, rehabilitation therapists, and nurses can all visit together. | Long-term nurse-patient relationships evolve into family-like bonds with emotional support.  Loyal patients envision expanded services, showing investment in platform success. | Quasi-family relationship  Vision for multidisciplinary care | From professional interaction to 'quasi-family' bonds  Engaged participation in services improvement | Affective dimension—deepening trust through emotional and cultural connection |
| P8: I need regular catheter changes and enteral feeding tube care. For these, as long as a nurse can come to my home to handle these issues, that's fine—convenience is key, and it doesn't matter who comes. However, when it comes to wound care, I want a specialist nurse to do it.  I hope it can be covered by basic medical insurance, reimbursing part of the cost, or by commercial insurance for full reimbursement. | Different care needs lead to different trust formation patterns.  Patients provide constructive feedback for service improvement. | Differentiated trust based on care complexity  Suggestions for insurance coverage | Personalized care to emotional comfort  Engaged participation in services improvement | N/A |
| P2: Nurse Li is like my daughter. She is professional in wound care, chats with me. | Emotional companionship is central to elderly patients' trust. | Emotional companionship for elderly | Personalized care to emotional comfort | N/A |
| P11: Every hospital visit in the past meant my offspring had to take turns using their precious leave to accompany me. It was hard on their jobs. The guilt would weigh on my conscience. Now, with the nurses coming, no one needs to take leave. Everyone is so much happier.  My offspring are abroad, I'm lying at home. They are very filial, although they can't be here to look after me... Now, they book nurses for me online. Every time a nurse visits, they join a video call and talk with her about my health. They feel much more at ease abroad. | Services alleviate burden on family, reducing patient guilt and building trust.  Digital home care enables distant offspring to fulfill filial duties, strengthening trust. | Alleviating family burden  Digital bridge for filial piety | Filial piety culture as emotional and trust catalyst  Filial piety culture as emotional and trust catalyst | N/A |
| P9: If there are long-term bedridden patients who need care, I will recommend it to them.  Once fully recovered and mobile, I won't need nurses to come home. | Loyal patients voluntarily promote services to peers with similar needs.  Patients recognize service discontinuation when needs are resolved. | Word-of-mouth recommendation  Service discontinuation when needs resolved | Trust-driven advocacy and word-of-mouth  Trust-based decisions to continue or terminate services | Conative dimension—translating trust into loyal behaviors within rational limits |
| P4: Home-based Care is especially beneficial for patients discharged after major surgery with weakened immunity. It saves them from going to the hospital and reduces cross-infection. I will recommend it to these people.  Unless my condition changes and I must be hospitalized, otherwise I will continue to choose it. | Recommendations are targeted based on specific patient needs and situations.  Trust and loyalty have rational boundaries based on clinical appropriateness. | Targeted recommendations  Conditional loyalty | Trust-driven advocacy and word-of-mouth  Trust-based decisions to continue or terminate services |  |
| P10: Each visit costs two to three hundred yuan, and after over 100 visits, the financial pressure is not small, but I still insist on choosing it. | Patients are willing to pay premium prices despite financial pressure due to trust. | Willingness to pay premium | Willingness to pay as a monetization of trust | N/A |
